# Formulation Development of a COVID-19 Recombinant Spike Protein-Based Vaccine

**DOI:** 10.3390/vaccines12080830

**Published:** 2024-07-23

**Authors:** Emily Xiao, Clémentine Mirabel, Didier Clénet, Shaolong Zhu, Andrew James, Luciano Ettorre, Trevor Williams, Jason Szeto, Nausheen Rahman, Salvador Fernando Ausar

**Affiliations:** 1 Global Vaccine Drug Product Development, Sanofi, 1755 Steeles Avenue West, Toronto, ON M2R 3T4, Canada; clementine.mirabel@gmail.com (C.M.); nausheen.rahman@sanofi.com (N.R.); 2 Global Vaccine Drug Product Development, Sanofi, 1541 Avenue Marcel Mérieux, 69280 Marcy-L’Étoile, France; didier.clenet@sanofi.com; 3 Analytical Sciences, Sanofi, 1755 Steeles Avenue West, Toronto, ON M2R 3T4, Canada; shaolong.zhu@sanofi.com (S.Z.); luciano.ettorre@sanofi.com (L.E.); trevor.williams@sanofi.com (T.W.); jason.szeto@sanofi.com (J.S.); 4 External Research and Development, Sanofi, 1755 Steeles Avenue West, Toronto, ON M2R 3T4, Canada; andrew.james@sanofi.com

**Keywords:** vaccine, COVID-19, SARS-CoV-2, spike protein, agitation stress, thermal stress, stability, HDX-MS

## Abstract

The purpose of this study was to develop a formulation for a recombinant prefusion spike protein vaccine against SARS-CoV-2. It was found that the spike protein was susceptible to aggregation due to mechanical stress. Therefore, formulation studies were initiated focused on screening pharmaceutical excipients capable of preventing this. The screening of a panel of potential stabilizing conditions found that Tween 20 could inhibit mechanically induced aggregation. A concentration-dependent study indicated that a higher concentration of Tween 20 (0.2% *v*/*v*) was required to prevent conformational changes in the trimer. The conformational changes induced by mechanical stress were characterized by size exclusion chromatography (SEC) and hydrogen–deuterium exchange mass spectrometry (HDX-MS), indicating the formation of an extended trimeric conformation that was also unable to bind to antibodies directed to the S2 domain. Long-term stability modeling, using advanced kinetic analysis, indicated that the formulation containing 0.2% (*v*/*v*) Tween 20 at a neutral pH was predicted to be stable for at least two years at 2 °C to 8 °C. Additional stabilizer screening conducted by thermal shift assay indicated that sucrose and glycerol were able to significantly increase the spike protein melting temperature (Tm) and improve the overall thermostability of the spike protein in a short-term stability study. Thus, while 0.2% (*v*/*v*) Tween 20 was sufficient to prevent aggregation and to maintain spike protein stability under refrigeration, the addition of sucrose further improved vaccine thermostability. Altogether, our study provides a systematic approach to the formulation of protein-based COVID-19 vaccine and highlights the impact of mechanical stress on the conformation of the spike protein and the significance of surfactants and stabilizers in maintaining the structural and functional integrity of the spike protein.

## 1. Introduction

The SARS-CoV-2 coronavirus spike protein is the most important viral envelope glycoprotein and mediates virus attachment and entry into host cells [1,2,3]. The spike protein is composed of two functional subunits: the S1 subunit is responsible for binding to the host cell receptor, while the S2 subunit mediates fusion of the viral and host cellular membranes [1,4]. The spike protein trimer is located on the surface of the virus, protruding as a mushroom-like structure where three S1 subunits are arranged to form a cap and three S2 subunits form the stalk [5]. The S1 subunit contains the receptor binding domain, which binds to the host cellular receptor, the Angiotensin Converting Enzyme-2 (ACE2), a membrane-bound carboxypeptidase localized to vascular endothelial and epithelial surfaces [5].

Costello et al. [6] described two conformational states of the native spike protein. The prefusion structure, resolved by cryo-electron microscopy, was referred to as the ‘canonical trimer’; and a low-temperature stabilized conformation, where epitope presentation in the S2 domain was altered, was referred to as the ‘open trimer’. Bruch et al. [7] further characterized the spike antigen conformations in both the ancestral (wild-type) and beta vaccine antigens developed by Sanofi and further discussed in this work.

The trimeric spike glycoprotein is the principal target of neutralizing antibodies and of most experimental and approved vaccines, including messenger RNA (mRNA), protein subunit, viral vector, and inactivated vaccines encoding the spike protein of SARS-CoV-2, which were shown to induce neutralizing antibodies conferring protection against severe disease [8]. The Sanofi antigen in the commercial Vidprevtyn^®^ vaccine is a stabilized prefusion trimer of the SARS-CoV-2 spike protein from the beta variant (B.1.351), which arose from the ancestral strain (D614) [9]. The vaccine antigen contains a mutation of the furin cleavage site and replacement of the transmembrane region by the T4 foldon trimerization domain. In addition, it contains double proline substitutions (2P) at the beginning of the central helix of the S2 subunit to stabilize the structure and prevent pre-fusion to post-fusion conformational changes in the trimer [10]. Undoubtedly, mRNA as well as vector-based vaccine strategies offered both faster time to the clinic and the capability to produce significant quantities of vaccine [11]. Nevertheless, the efficacy of the mRNA approved vaccines is significantly waning considering the evolution of the virus, giving rise to novel variants of concern. Recombinant protein vaccines offer distinct benefits over the nucleic acid or viral-vector vaccines, such as an established history of safety and efficacy, and relatively higher stability compared to mRNA and viral vector vaccines, which facilitate production and distribution in low- and middle-income countries [12].

The formulation development of recombinant protein-based vaccines still requires several steps to ensure the design of the formulation is robust enough to endure the many stresses encountered during handling, manufacturing, storage, and transportation. One of the primary challenges in developing protein-based vaccines is identifying the optimal formulation. Excipients and stabilizers are meticulously screened to maintain the protein conformation and chemical integrity. To assess the stability of the formulated vaccine, accelerated stability studies are conducted by exposing the vaccine to conditions that simulate the stresses it might encounter during its shelf life, such as high temperature and agitation stress. By monitoring the protein antigen’s integrity, purity, and potency over time, valuable insights can be gained into the vaccine’s stability profile and predict its shelf life under different storage conditions.

COVID-19 vaccines contain various formulation buffers and stabilizers to ensure their stability. These components help maintain the vaccine’s integrity during storage and transportation. In the case of mRNA-LNP vaccines, in addition to buffers and NaCl that help maintain pH and tonicity, sucrose is added to protect the nanoparticle structure during freezing and thawing [13]. Another common ingredient found in viral vector vaccines (e.g., AstraZeneca, Johnson & Johnson) and protein subunit vaccines (e.g., Novavax) is polysorbate 80, which is added to prevent viral vector or protein aggregation [14].

In this manuscript, we provide a thorough account of the formulation optimization of a SARS-CoV-2 spike antigen. The formulation development was initiated with the ancestral spike protein and later applied to the beta spike protein used as a booster vaccine. By carefully examining the role of Tween 20, the impact on trimer conformation, in vitro potency, and predicted shelf life, we offer essential insights into the formulation’s critical parameters. Additionally, our exploration of supplementary excipients could play a pivotal role in fine-tuning the formulation to enhance its robustness during storage and transportation, which is of the utmost importance to vaccine and therapeutic development.

## 2. Materials and Methods

### 2.1. Materials

The prefusion spike protein with deleted transmembrane domain either from the SARS-CoV-2 ancestral variant (D614) or from the beta variant (B.1.351) were expressed in baculovirus and then purified by chromatography and buffer exchanged in 10 mM Phosphate Buffer Saline (PBS) pH 7.0 with or without 0.02% Tween 20 (*v*/*v*). Experiments evaluating the effect of excipients were conducted with spike protein concentrations levels of the drug substance (DS) stage (150 to 190 µg/mL) or the drug product (DP) stage (20 to 60 µg/mL) [7].

The following excipients were used during buffer and excipient screening: sodium phosphate monobasic and dibasic, sodium acetate, sodium chloride, heparin, sucrose, Zwittergent 3–10, Zwittergent 3–12, and 0.1% Zwittergent 3–14 were all purchased from MilliporeSigma (Saint Louis, MO, USA). Glycerol (Invitrogen, Waltham, MA, USA), Tween 80 (Emprove^®^, Millipore Sigma, Saint Louis, MO, USA), Tween 20 (JT Baker, Phillipsburg, NJ, USA), and 0.5 M Tris buffer pH 8.0 (Alfa Aesar, Haverhill, MA, USA) were also assessed.

### 2.2. Agitation Stress Studies

The ancestral spike antigen (D614) was utilized to screen excipients that can potentially prevent aggregation. The ancestral spike antigen in different buffering conditions was prepared at 170 µg/mL and filled into 3 mL glass vials at 0.68 mL/vial. The vials were placed securely on a horizontal fashion orbital shaker (VWR model 3500, Radnor, PA, USA) at ambient temperature and agitated at 300 rpm for 24 h as previously described [15]. The samples were evaluated by testing turbidity and ACE2 binding before and after agitation in the presence of potential stabilizers. In experiments evaluating the effect of Tween 20 concentration, in vitro potency by enzyme-linked immunosorbent assay (ELISA), open trimer peak area by size exclusion chromatography-ultra-performance liquid chromatography (SEC-UPLC), ACE2 binding and anti-S2 binding were tested before and after agitation.

### 2.3. Far UV Circular Dichroism and Intrinsic Tryptophan Fluorescence Spectrometry

Far UV circular dichroism (CD) spectra and intrinsic fluorescence spectra were collected using a Chirascan Plus spectrophotometer (Applied Photophysics, Surrey, UK) equipped with fluorescence detector, a Peltier temperature controller, and an automated four-position cuvette holder as previously described [15]. To obtain the CD spectra, diluted spike protein solutions were prepared at a protein concentration of 150 µg/mL and placed in 0.1 cm path length quartz cuvettes. Spectra were acquired from 195 to 300 nm using a 1 nm spectral resolution, a scanning speed of 0.5 s per data point, and a 1 nm bandwidth. Intrinsic tryptophane fluorescence emission spectra were obtained in 1 cm path length quartz cuvettes using excitation at 290 nm and a 1 nm bandwidth. The fluorescence spectra were collected from 280 to 450 nm at 1 nm resolution.

To quantify spectral differences before and after stress studies, a weighted spectral difference (WSD) calculation was used [16,17,18]. The WSD reflects the dissimilarity between CD or fluorescence spectra, and then quantitatively informs on higher-order structure (HOS) differences between samples or HOS changes in antigen during stresses. In general, WSD approaches zero for identical spectra and increases with the increase in differences between spectra.

$$WSD=\sqrt{\frac{1}{n}\sum_{i=1}^{n}\frac{\left|A_{i}\right|}{\left|\bar{A}\right|}{\left(A_{i}-B_{i}\right)}^{2}}$$

where *A* and *B* are absorbance values of the reference and sample spectra, respectively, with n data points in a spectral range of interest.

### 2.4. Extrinsic Fluorescence Studies

Thermal ramping studies to obtain the denaturation Tm of the spike protein were conducted by extrinsic fluorescence as described elsewhere [19]. Briefly, a Mx3005p instrument (Stratagene, La Jolla, CA, USA) was employed to detect the unfolding process of the protein antigen. The spike protein was diluted in the different buffering conditions in a range of 140 to 170 µg/mL and the unfolding was detected by introducing the extrinsic dye SYPRO Orange (Invitrogen, Carlsbad, CA, USA). A sample volume of 100 µL was loaded to 96-well polypropylene plates and capped with optical cap strips (Stratagene, La Jolla, CA, USA) to prevent sample evaporation. The plates were heated at 1 °C/min from 25 °C to 96 °C. The instrument’s excitation and emission filters were set at 492 and 610 nm, respectively, and the fluorescence at 610 nm (excitation 492 nm) was collected every 1 °C. Melting temperatures were obtained by calculating the minimum of the negative first derivative plot of the heating trace.

### 2.5. Thermal Stability Studies

Spike protein at the DS stage or at the DP stage in the different buffering conditions were prepared and filled into glass vials and incubated for various times at 5 °C, 25 °C, 37 °C, and/or 41 °C. For the short duration thermal stress stability studies, the vials were incubated in an inverted position at 37 °C for 7 to 15 days.

The stability of the spike protein was evaluated as a function of time by measuring the peak area of trimeric forms by SEC-UPLC, in vitro potency by ELISA, and ACE2/anti-S2 binding, as described below.

### 2.6. Advanced Kinetic Modeling for Stability Prediction

The kinetic analysis of the stability data was performed using AKTS—Thermokinetics software (version 5.51, AKTS AG, Advanced Kinetics Technology Solutions, Siders, Switzerland) as previously described [20,21]. Briefly, various Arrhenius-based models, from simple to more sophisticated ones, are screened to fit short-term in vitro ELISA potency stability data obtained at typical storage temperature (5 °C) and higher temperatures (25 °C, 37 °C, and 41 °C). The simplest model best describing the progress of the change in the considered attribute is then selected using statistical scores such as weighted Akaike and Bayesian Information Criteria (wAIC/wBIC). When the wAIC and wBIC criteria identified two different models, both were considered to perform multiple bootstraps. Using this multiple model bootstrap (MMB) approach, a bootstrap was made on a single model, with the number of loops (Nloops) being proportional to its respective values of the weights wAIC and wBIC, then leading to a one-sided lower 95% prediction interval (PI). The design of the experiments ensured that the modeling practices recommended for accelerated stability predictions of bioproducts were followed [22,23,24]. Long-term predictions of the stability of potency data from the in vitro potency measured by ELISA for products stored under the recommended storage condition (5 °C), including a short excursion at ambient temperature, were also determined using the selected models and corresponding PIs.

### 2.7. Physical Appearance and pH

Physical appearance and pH were recorded for every sample recovered from the thermal stability studies as a routine monitor for gross abnormalities. Physical appearance was examined for color, presence of fibers, change in uniformity, and particulates/aggregation/precipitates. The test was performed visually under normal laboratory light or extra light with the assistance of a magnifying glass in white or black background settings. The pH was measured using Mettler Toledo Seven Excellence Multiparameter S500 pH Meter.

### 2.8. Size Exclusion Chromatography (SEC)

SEC was performed on an ultra-high-pressure and low dispersion liquid chromatographic system (UPLC, Agilent 1290 Infinity II Bio, Santa Clara, CA, USA), equipped with an ultraviolet (UV) detector and sub-2 µm particle size column with 200 Å pores (column from AdvanceBio SEC 200A 1.9 µm 4.6 × 300 mm S.N. 0006514605-17). An isocratic aqueous mobile phase (0.01 M sodium phosphate with 0.3 M sodium chloride, pH 7.0) was used to achieve high resolution separation of the CoV-2 trimeric and monomeric spike protein forms from process impurities and degradants in 15 min. A mixture of molecular weight standards was run for reference, and proteins and impurities were detected by UV absorbance at 215 nm. The samples were centrifugated to remove potential large aggregates prior injection and the injection volume was based on the spike protein concentration with total 0.29 µg protein injection (e.g., 4.8 µL of 60 µg/mL sample and 7.2 µL of 40 µg/mL).

The chromatogram was integrated, and % area values were monitored for the three main trimeric peaks. The following trimer peaks were obtained: an open trimer, the native trimer peak observed in samples stored at 2 °C to 8 °C, an extended trimer, the earlier eluting peak induced by thermal or mechanical stress (extended trimer), and a canonical trimer, the later eluting peaking induced by thermal stress (Supplementary Figure S1). The data for open trimer peak area (SEC-UPLC) and potency (ELISA) correlated well for the samples containing the same formulation under the forced degradation temperature (Supplementary Figure S3).

### 2.9. In Vitro Potency Measured by ELISA

An in vitro potency test using ELISA was performed on monovalent SARS-CoV-2 B.1.351 and D614 spike protein samples at the stages of unadjuvanted DS and final DP. First, 96-well microtiter plates were coated with a monoclonal antibody (mAb) specific to the S2 domain of SARS-CoV-2 spike proteins (Sanofi mAb “875”) in order to capture the spike antigen present in the sample. Following sample capture, the monovalent D614 Spike antigen sample was detected by addition of a neutralizing mAb specific to the S1 domain of the D614 spike protein (mAb “886”) conjugated with horseradish peroxidase (HRP), while the monovalent B.1.351 spike protein sample was detected by the addition of a neutralizing mAb that bound the S1 domain of the B.1.351 spike protein (mAb 876) conjugated to the HRP. The addition of the substrate tetramethylbenzidine (TMB) causes the enzyme (HRP) to catalyze the reaction, converting the TMB into a colored compound, and the reaction was stopped by the addition of 2 M H_2_SO_4_. The ELISA plates were read by measuring the absorbance of each well at a wavelength of 450 nm with a reference wavelength of 540 nm. The ELISA data were analyzed using SoftMax Pro version 6.5.1 GxP software using a 4-PL model. Parallelism was assessed using the equivalence test approach of the slope ratio, whereby the confidence intervals of the test article must be within set equivalence limits in order to prove parallelism with the respective reference standard. Relative potency was determined based on comparison to the reference standard and used to calculate a reportable value for sample reported as ‘ELISA test units’ per mL (ETU/mL).

### 2.10. ACE2 Binding and Anti-S2 Binding Assays

Surface Plasmon Resonance (SPR) was applied to monitor the ability of D614 spike protein to bind to purified human ACE2 receptor (ACE2-Fc-Avi-His, Sydlabs, Hopkinton, MA, USA), and the ability of the spike protein S2 domain to be recognized by an anti-S2 mAb (mAb clone 511; Abcellera Inc., Vancouver, BC, Canada) [25] that binds to a conformational S2 epitope. The SPR assays were carried out as previously described [7].

### 2.11. Turbidity

Turbidity measurements were carried out to detect protein aggregation during the screening for excipients for protection against agitation stress and thermal stress. The turbidity measurements were carried out in the Core Module 3 (CM3) automated robot using the visual station attachment (Unchain Labs, Sunnyvale, CA, USA). A series of commercially available standards with Nephelometric Turbidity Units (NTUs) ranging from 0 to 1000 NTUs were used as calibration standards.

### 2.12. Hydrogen Deuterium Exchange Mass Spectrometry (HDX-MS)

Prior to conducting the HDX-MS experiments, the spike protein (pre- and post-agitation) was buffer exchanged into 10 mM phosphate buffer using detergent removal spin columns to minimize the presence of Tween detergent in the sample.

The HDX-MS experiments were conducted as described in [7] with minor modifications. The differences were as follows: the Buffer E contained 10 mM phosphate buffer, 150 mM NaCl at pH 7.5 and the labeling buffer is deuterated Buffer E with a pD of 7.5. Five HDX-MS timepoints (20 s, 2 min, 10 min, 30 min, and 60 min) were acquired in technical triplicates. The guard and analytical columns, liquid chromatography (LC) gradient, and mass spectrometer (MS) acquisition parameters were same as in [7]. Briefly, the non-deuterated and deuterated samples were quenched with subsequent digestion in the nano ACQUITY UPLC HDX-MS module housing a (1:1) pepsin/protease XIII (NovaBioAssays, MA, USA) enzymatic column at 15 °C. The resulting digested peptides were desalted using a Waters BEH C18 guard column (100 µL/min for 3 min) and separated using a Waters CSH C18 analytical column (30 µL/min using 7 min gradient) at 0 °C. The eluted peptides were electrosprayed and detected using Waters Synapt G2-Si mass spectrometer using GluFib (785.8426 *m*/*z*) as a lock mass solution to maintain mass calibration of <10 ppm.

Peptide identification was performed by using ProteinLynx Global Server software v3.0.2 (Waters Corp., Milford, MA, USA) and deuterium exchange analysis was performed on DynamX software v3.0.0 (Waters Corp., Milford, MA, USA). If the differences exceeded 2.0 Da and surpassed three times the standard deviation, they were considered statistically significant.

### 2.13. Statistical Analysis

Simple linear regression was used to assess the effect of the Tween 20 concentration on the thermal stability at 37 °C of the spike protein at 170 µg/mL and 60 µg/mL using GraphPad Prism 9 software (Boston, MA, USA). The software compared the slopes of two or more regression lines and this method is equivalent to an analysis of covariance (ANCOVA) [26].

## 3. Results

### 3.1. Screening of Excipients That May Prevent Mechanical Stress Induced Aggregation of Spike Protein

**Figure 1:**
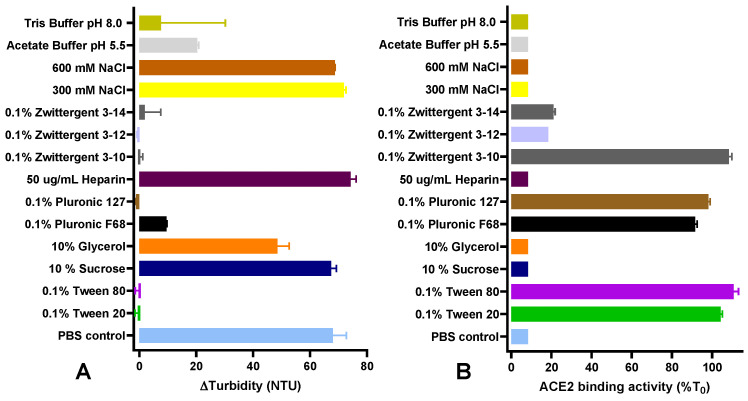
Screening of potential excipient to protect spike protein (D614) against mechanical stress. Spike protein at 170 µg/mL was subjected to mechanical stress at ambient temperature in an orbital shaker at 250 RPM for 24 h. Turbidity (**A**) and ACE2 binding activity (**B**) was measured before and after mechanical stress (*n* = 2). Turbidity is presented as Delta turbidity, which corresponds to the turbidity measured at 24 h of mechanical stress minus the turbidity obtained at time zero. ACE2 binding activity is presented as percentage of activity obtained at 24 h of mechanical stress with respect to the activity obtained at time zero. Unless otherwise noted, all experiments were performed in 10 mM PBS pH 7.0.

Protein aggregation is a commonly encountered and challenging phenomenon in vaccine development. To address this concern, formulation studies focused on the screening of pharmaceutical excipients capable of preventing the aggregation of spike protein candidates [27,28]. Mechanical stress is often applied in these studies to increase the exposure of the proteins to the air–liquid interface [29]. This not only exacerbates aggregation but also simulates the conditions that the vaccine may encounter during manufacturing and transportation. Thus, to screen for pharmaceutical acceptable excipients that could prevent D614 spike protein aggregation, the protein was subjected to mechanical stress for 24 h at 300 rpm in glass vials and tested for turbidity and the ability of the spike protein to bind to the ACE2 receptor. In the control formulation containing PBS at pH 7.0, after 24 h of mechanical stress, a change in the physical appearance was observed. The initially clear colorless solution observed at time zero (T_0_) turned into a turbid solution with visible particles, displaying a significant increase in turbidity (Figure 1A). After 24 h of agitation, the formulations containing Tween 20, Tween 80, Pluronic 127, Zwittergent 3–12, and Zwittergent 3–14 displayed minimal or no changes in turbidity (Figure 1A). In good agreement, the ACE2 binding activity was maintained when Tween 20, Tween 80, Pluronic 127, or Zwittergent 3–10 were added to the formulation (Figure 1B). However, a significant decrease in the ACE2 binding activity was detected in formulations containing Zwittergent 3–12 and Zwittergent 3–14, suggesting that these compounds were not able to protect the spike protein against mechanical stress, at least by maintaining the correct conformation of the receptor binding domain (Figure 1B). An alternative explanation for the decreased ACE2 binding is that these surfactants might bind to the protein at sites that hinder ACE2 binding.

### 3.2. Tween 20 Increases the Robustness of Spike Protein against Mechanical Stress in a Concentration-Dependent Manner

**Figure 2:**
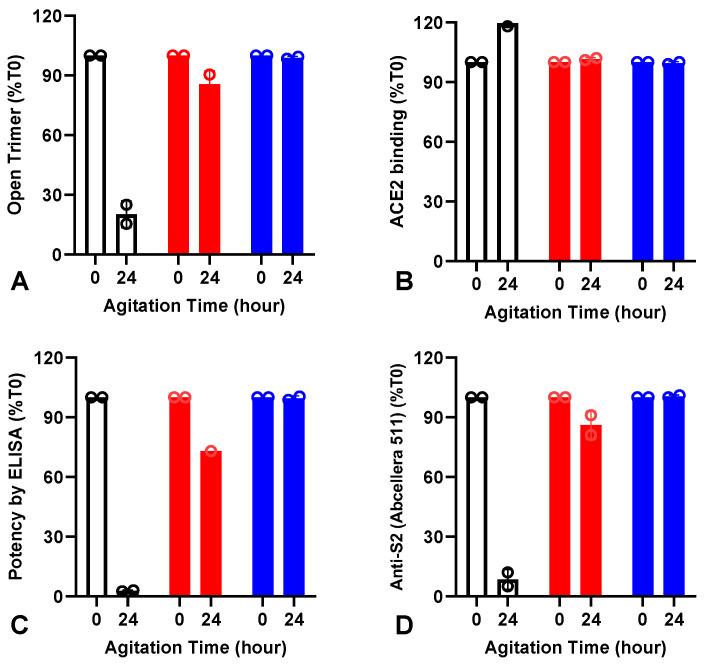
Effect of mechanical stress on spike protein (D614) containing increasing concentrations of Tween 20 in 10 mM PBS pH 7.0. Spike protein (D614) at a concentration of 60 µg/mL with 0.02% Tween 20 (white bar), 0.1% Tween 20 (red bar) and 0.2% Tween 20 (blue bar) was subjected to agitation stress in an orbital shaker at 300 rpm for 24 h and tested for open trimer area by SEC-UPLC (**A**), ACE2 binding (**B**), in vitro potency measured by ELISA (**C**), and anti-S2 binding (Abcellera 511) (**D**). All results are presented as percentage of signal obtained after 24 h of mechanical stress relative to the signal obtained at time zero (%T_0_) (*n* = 2).

From the list of excipients that were able to prevent mechanically induced aggregation and losses in the ACE2 binding, the surfactant Tween 20 was selected for further optimization. The reason for selecting Tween 20 was that it is a generally regarded as a safe ingredient commonly found in vaccines and, in addition, it was an excipient utilized during the downstream purification process. We thus investigated the robustness of the spike protein (D614) against mechanical stress in formulations with increasing concentrations of Tween 20 ranging from 0.02% to 0.2% (*v*/*v*). At time zero (T_0_) and after 24 h of agitation, the robustness of the spike protein against mechanical stress was assessed using SEC-UPLC, ACE2 receptor binding, anti-S2 binding activity, and in vitro potency using ELISA (Figure 2). At a low concentration of Tween 20 (0.02% *v*/*v*) and after 24 h of mechanical stress, the SEC-UPLC chromatograms showed a shift in the trimeric peak to an extended trimer at the expense of a significant decrease in the peak area of the open trimer (Supplementary Figure S2A and Figure 2A). The shift in the trimeric peak in the SEC-UPLC chromatograms observed with a low concentration of Tween 20 correlated well with a decrease in the in vitro potency ELISA results (Figure 2C) and anti-S2 (Abcellera 511) binding activity (Figure 2D), with a small increase in the ACE2 binding activity (Figure 2B). When the concentration of Tween 20 was increased to 0.1%, the mechanical stress induced a small but measurable decrease in the open trimer peak area, potency, and anti-S2 (Abcellera 511) binding activity (Figure 2A,C,D), while no changes were observed in the ACE2 binding (Figure 2B). At the highest concentration of Tween 20 tested (0.2%), the mechanical stress exhibited no discernible impact on the spike protein, as illustrated by the absence of changes in any of the measured quality attributes (Figure 2A–D). These findings strongly suggest that a minimum concentration of 0.2% Tween 20 is necessary to effectively prevent mechanical-stress-induced changes in the spike protein. The effect of Tween 20 on the conformation of the spike protein was confirmed with the beta variant (B1.351) (Supplementary Figure S2C). At a high concentration of Tween 20, it is plausible that the surfactant forms a protective barrier around the spike protein and the air water interface, preventing conformational changes upon exposure to mechanical stress. The surfactant may also prevent protein-to-protein interaction, reducing the hydrophobic effect of hydrophobic patches on the surface of the protein.

### 3.3. The Structural Basis of Tween 20 Protection for the Spike Protein against Mechanical Stress

To further investigate the impact of Tween 20 on the conformation of the ancestral spike protein strain (D614) under mechanical stress, we examined changes in the secondary and tertiary structure of the protein using circular dichroism and fluorescence spectroscopy, respectively. At a low concentration of Tween 20 (0.02% *v*/*v*), after 24 h of agitation, the CD spectrum of the spike protein showed a moderate decrease in both the negative peak at 208 nm and the shoulder at 220 nm, suggesting a loss in alpha-helical structure (Supplementary Figure S3B). On the contrary, the formulation containing a high concentration of Tween 20 (0.2% *v*/*v*) showed no changes in the CD spectrum after agitation, displaying an almost identical CD spectrum to that of the non-agitated control (Supplementary Figure S4A). When examining the fluorescence spectrum, the formulation containing 0.02% Tween 20 (*v*/*v*) showed minor but reproducible changes in the emission spectrum of the spike protein. Although the peak maximum at 335 nm remained unchanged, the fluorescence intensity reduced after agitation. Additionally, the appearance of a small peak at the same excitation wavelength indicated an increase in light scattering, suggesting the formation of higher molecular weight species upon agitation (Supplementary Figure S4B). In good agreement with the CD results, the fluorescence emission spectra of the formulation in 0.2% Tween 20 (*v*/*v*) showed no significant changes upon agitation (Supplementary Figure S4A). The effect of the agitation stress on the spike protein (D614) was also further estimated by the WSD of the CD spectra and intrinsic fluorescence spectra and a significant difference in WSD was found for the formulation containing 0.02% (*v*/*v*) Tween 20 and 0.2% (*v*/*v*) Tween 20 (Supplementary Figure S4C). The findings collectively suggest that Tween 20 at 0.2% (*v*/*v*) concentration effectively prevents changes in both the secondary and tertiary structure of spike protein subjected to mechanical stress.

**Figure 3:**
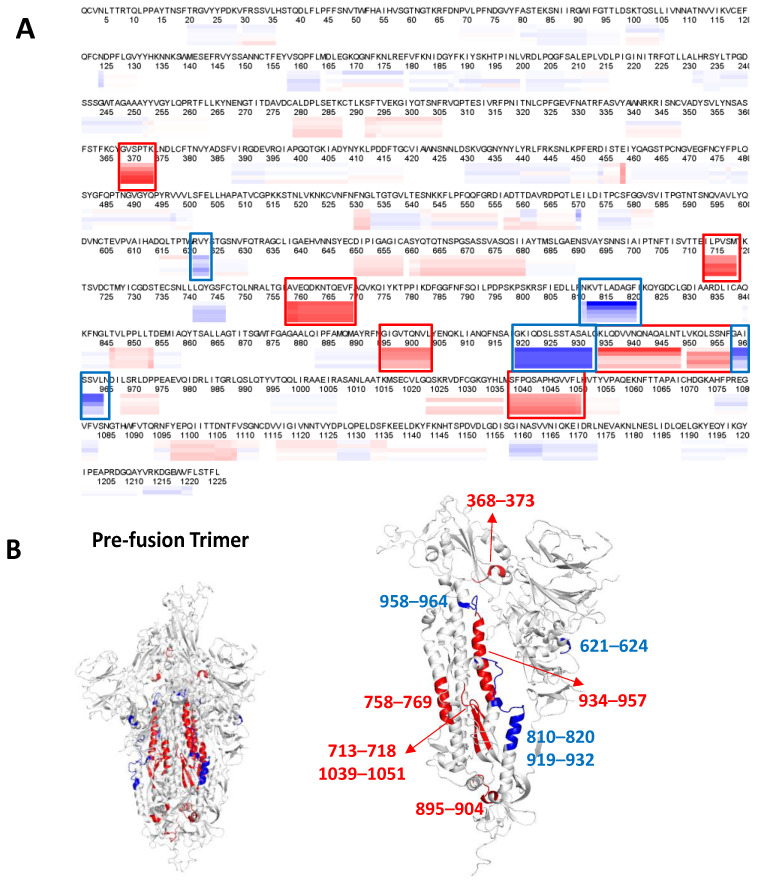
(**A**) Dynamic analysis of spike protein (D614) pre- and post-agitation in 0.02% Tween formulation. The data are represented as fractional uptake view where red indicates increase in deuterium uptake and blue represents decrease in deuterium uptake when the sample was agitated. The degree of magnitude is shown in the blue–red spectrum. The regions that showed changes are shown in red and blue boxes. (**B**) The HDX-MS data were mapped onto monomeric view of the pre-fusion structures (PDB: 6XM3). The inset shows the trimeric view.

To gain further insight into the conformational and/or structural changes in the spike protein when subjected to mechanical stress, we employed HDX-MS to understand how agitation stress affected the conformation of the spike protein. HDX-MS studies were conducted on spike protein ancestral strain (D614) samples containing 0.02% (*v*/*v*) Tween 20 since, under this condition, the CD and fluorescence experiments exhibited significant spectral changes (Supplementary Figure S4), thereby enhancing the likelihood of detecting structural alterations to the protein. Native and agitated samples of the spike protein were compared. Based on the HDX-MS heatmap analysis, as shown in Figure 3A, differences in deuterium uptake were detected when the spike protein was agitated. These changes were mainly localized in the S2 stalk region, with minimal impact on the receptor binding domain and S1 domain. Residues 713–718, 758–769, 895–904, 934–957, and 1039–1051 showed an increase in deuterium uptake, whereas residues 621–624, 810–820, 919–932, and 958–964 showed a decrease in deuterium uptake. Residues 368–373 of the S1 domain were also affected where there was an increase in deuterium uptake when agitated. Figure 3B shows the same HDX-MS data mapped onto the three-dimensional structure of the pre-fusion spike protein (D614). A majority of the residues that exhibited increased deuterium uptake upon agitation were localized in the hydrophobic core of the S2 domains (trimeric interface region) of the pre-fusion trimeric structure, sequestered from the solvent, whereas the residues that showed a decrease in deuterium uptake were localized in the solvent-exposed region. The increases in deuterium uptake could indicate more solvent exposure in these regions or disruption of the hydrogen bonding network, while the decreases in deuterium uptake were likely the result of diminished solvent exposure or of the enhanced hydrogen bonding network formed to stabilize the secondary structure. The HDX-MS results indicate that the conformational changes induced by agitation were primarily in the S2 domain. Spike protein is known to adopt an open trimeric form when stored at 2 °C to 8 °C [6]. Based on the HDX-MS analysis (shown in Figure 3B), the S2 stalk regions exhibited increases in deuterium uptake, which could indicate further extension/expansion of the trimeric conformation. The interpretation of the HDX-MS data supports the SEC-UPLC analysis of the agitated spike protein, where an early eluting species, implying an increase in the Stokes radius (extended trimer, Figure 2A), was observed. Furthermore, the minimal changes in deuterium uptake in the S1 domain, indicative of a stable structure for the receptor binding domain, were also supported by consistent ACE2 binding for both the native and agitated samples, as shown in Figure 2B (0.02% Tween 20).

### 3.4. Impact of Tween 20 on Spike Protein (D614) Thermal Stability

**Figure 4:**
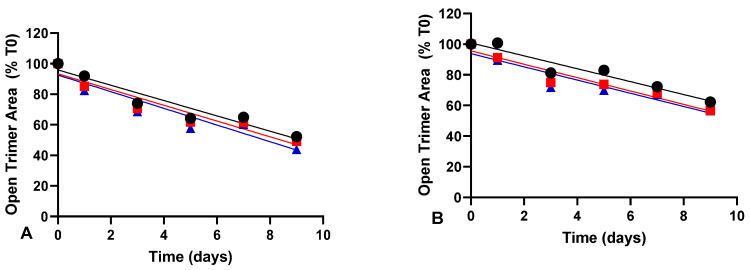
Effect of Tween 20 concentration on the thermal stability at 37 °C of ancestral spike protein (D614) at 170 µg/mL (**A**) and at 60 µg/mL (**B**). Formulations containing 0.02% Tween 20 (black circles), 0.1% Tween 20 (red squares), 0.2% Tween 20 (blue triangles) were incubated at 37 °C and the trimeric conformation of the spike protein was measured at different timepoints by SEC-UPLC. The results are presented as native trimer area as a percentage of time zero. No significant differences in the zero-order rate constant were obtained by ANCOVA when comparing the different concentration of Tween 20 for 170 µg/mL spike protein (*p* = 0.918) and 60 µg/mL spike protein (*p* = 0.985).

The mechanical stress studies shown in the above sections clearly demonstrated that 0.2% (*v*/*v*) Tween 20 effectively maintained the functional and structural integrity of spike protein subjected to mechanical stress. Subsequently, we aimed to investigate whether Tween 20 had any impact on the thermal stability of the spike protein. For this purpose, we examined the effect of increasing concentrations of Tween 20 on the ancestral version of the spike protein (D614) subjected to elevated temperature conditions and assessed the open trimer peak area by SEC-UPLC over time. When the spike protein was incubated at 37 °C, a quasi-linear downward trend was observed for all three concentrations of Tween 20 under evaluation (Figure 4). To simplify the analysis and considering a low period of time (i.e., 9 days at 37 °C), we operated under the assumption of zero-order kinetics and then compared the trends using ANCOVA. Similar downward trends were noted for both the 170 µg/mL spike protein DS and the 60 µg/mL spike protein (DP) (Figure 4). The results indicated that increasing the concentration of Tween 20 from 0.02% to 0.2% had no significant effect on the zero-order rate constant obtained at 37 °C (ANCOVA, *p* = 0.918 for 170 µg/mL spike protein and *p* = 0.985 for 60 µg/mL spike protein).

### 3.5. Stability Modeling for Drug Products Using In Vitro Potency Measured by ELISA Predicts at Least 12-Month Stability at 5 °C Storage

To gain better insight into the thermal stability and the shelf life of the selected formulation conditions, a stability study under normal and accelerated temperature conditions was initiated on scale-up batches of the spike protein strains D614 and B.1.351 DPs. The study was designed to generate informative data points aligned with the use of advanced kinetic modeling (AKM) (i.e., three different incubation temperatures and significant change observed in the more drastic conditions).

**Figure 5:**
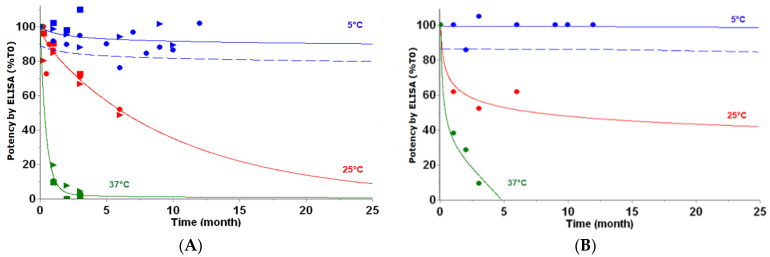
Long-term in vitro potency measured by ELISA for D614 variant at 40 µg/mL ((**A**) Three scale-up batches with Lot 1 as circles, Lot 2 as triangles, and Lot 3 as squares) and B.1.351 variant at 20 µg/mL ((**B**) one scale-up batch as circles) in formulation containing 0.2% Tween 20 predicted by kinetic models (lines) at 5 °C (blue), 25 °C (red), and 37 °C (green). Data used for kinetic modeling are displayed as filled symbols. At 5 °C, ELISA prediction is shown with predictive band representing 1-sided lower 95% PB (dashed lines).

These stability data for up to 12 months, considered sufficient to support clinical trials, were used to develop the kinetic models, leading to a two-step model for the D614 variant and to a mixture of a one-step and a two-step model for the B.1351 variants (Supplementary Table S1). These models suggested long-term stability with at least 80% of remaining potency (measured by ELISA) predicted for both variants over two years at 5 °C (Figure 5). These stability modeling results suggested that the selected formulation could maintain the stability of both variants, allowing for a shelf life that meets the desired product target profile.

### 3.6. The Thermal Stability of Spike Protein (D614) Is Increased by Sucrose and Glycerol

**Figure 6:**
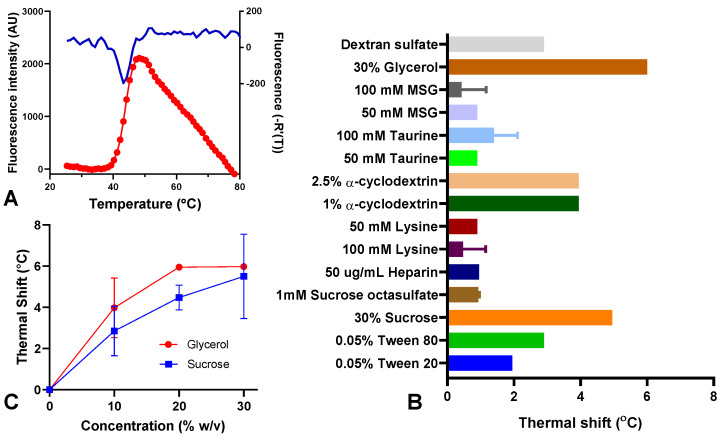
(**A**) Representative extrinsic fluorescence heating trace (red circles) and the first derivative (blue line) for spike protein (D614) in PBS 0.02% Tween 20, pH 7.0. (**B**) Screening of potential stabilizers of spike protein (D614) by thermal shift assay. The effect of the stabilizers on the stability of spike protein is plotted as follows: Thermal shift = T_m_ (Stabilizer) − T_m_ (Control). (**C**) Effect of the concentration of glycerol and sucrose on the thermal shift in spike protein. Data are presented as mean ± standard deviation (*n* = 2).

Having established that the colloidal stability and robustness against mechanical stress of the spike protein were improved by Tween 20, we proceeded to investigate whether the thermal stability could be further increased. To this end, a screening of potential stabilizers was performed by measuring their ability to enhance the conformational stability of the spike protein using extrinsic fluorescence spectroscopy. This method is based on detecting the increase in fluorescence intensity of the dye SYPRO Orange when bound to hydrophobic areas of thermally unfolded proteins. By monitoring the fluorescence intensity as a function of temperature, the melting point of the spike protein can be detected, as shown in Figure 6A.

As shown in Figure 6B, a total of 11 excipients were evaluated by their capacity to increase the Tm of the spike protein (D614). A positive shift in the Tm was observed for most of the excipients, indicating an increase in protein conformational stability. Among these excipients, glycerol, sucrose, and cyclodextrin showed the highest thermal shift (Figure 6B). The two compounds selected for further investigation were glycerol, associated with a 6 °C increase in the Tm, and sucrose, showing an increase of 5 °C in the Tm at a 30% (*w*/*v*) final concentration. We investigated the effect of the concentration of sucrose and glycerol on the Tm to determine the optimal concentration for these two excipients in the DP. As shown in Figure 6C, the Tm achieved a phase plateau at 20% final concentration of glycerol while the Tm using sucrose showed a maximum value at 30%. Therefore 20% glycerol and 30% sucrose were selected for further evaluation in short-term high-stress studies.

**Figure 7:**
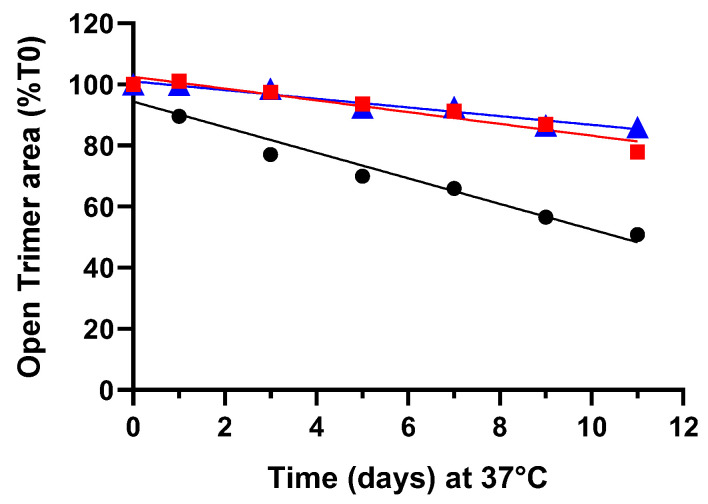
Effect of 20% glycerol and 30% sucrose on the thermal stability at 37 °C of spike protein (D614) at 60 µg/mL. Formulations containing 0.2% Tween 20 in PBS (black circles), 0.2% Tween 20 plus 20% glycerol in PBS (red squares), 0.2% Tween 20 plus 30% sucrose in PBS (blue triangles) were incubated at 37 °C and the trimeric conformation of the spike protein was measured at different timepoints by SEC-UPLC. The results are presented as native trimer area as a percentage of time zero. Extremely significant differences in the zero-order rate constant were obtained by ANCOVA when comparing the different concentration of Tween 20 for 60 µg/mL spike protein (*p* < 0.0001).

When examined by SEC-UPLC, we observed that formulations in the presence of sucrose or glycerol exhibited significantly higher stability compared to formulations in the absence of sucrose or glycerol at 37 °C ± 2 °C, based on the open trimer area data (Figure 7).

We then assessed the long-term stability prediction in the presence of sucrose as a stabilizer. We selected sucrose for the study because it is generally regarded as a safe stabilizer for parental products and commonly used in vaccines.

To evaluate the impact of sucrose on the long-term stability of the vaccines, an accelerated stability study including incubations of samples from 5 °C to 41 °C was performed for formulations containing 0.2% Tween 20 with 30% sucrose and compared to the control without sucrose (Supplementary Figure S5). AKM was applied to describe the loss of in vitro potency (measured by ELISA) of the spike protein (D614) as function of time, temperature, and the presence of sucrose, leading to a two-step kinetic model (Supplementary Table S1C). While the sucrose-based formulation induced a positive impact on the stability of the spike protein at elevated temperatures (Supplementary Figure S5), a comparable gradual decrease in the in vitro potency was predicted at 5 °C over one year for both formulations (Supplementary Figure S4). To illustrate this point, time–temperature–transformation (TTT) diagrams were plotted using the selected two-step models (Supplementary Figure S5). The isoconversion lines depict a change in slope of around 37 °C for both formulations, suggesting two different kinetics (reaction rates) below and above this temperature. Furthermore, while the isoconversion lines exhibit higher slopes for the sucrose-based formulation, indicating a thermo-stabilizing effect of this excipient, the time taken to reach a specific level of degradation (e.g., 20% loss of in vitro potency as illustrated by isoconversion lines at 0.2 seen in Supplementary Figure S5) looks similar for both formulations. This time to reach degradation is reduced at higher temperatures, especially above 37 °C, confirming a thermo-stabilizing effect of sucrose, mainly at high temperature. This property can be very useful in case of unexpected excursions of temperature for vaccines during storage or shipping, where sucrose could help maintain vaccine intactness.

## 4. Discussion

In this manuscript, we elucidate the intricate process of formulating a protein-based COVID-19 vaccine centered on the viral spike protein. An essential consideration in this formulation development was the protein’s susceptibility to aggregation under mechanical stress, prompting the screening of conditions that could mitigate this challenge. It was found that Tween 20 at 0.02% effectively prevented spike protein aggregation. However, it became evident that while this detergent concentration was successful in stopping aggregation, it fell short in preventing conformational changes that had a significant impact on the protein’s in vitro potency values. A tenfold higher concentration of Tween 20 was required to prevent conformational changes in the spike protein.

To gain a more comprehensive insight into the structural alterations of the spike protein under agitation, we employed CD and fluorescence spectroscopy. Notably, the spike protein exhibited conformational changes in both the secondary and tertiary structure exclusively in formulations containing 0.02% Tween 20. These findings suggest that, at a low concentration of Tween 20, which is below the critical micelle concentration (CMC), interfacial events at the air–water interface may trigger conformational changes in the spike protein trimeric structure. However, when the concentration of Tween 20 exceeded the critical CMC, the surfactant may have prevented conformational changes by impeding the interaction between the spike protein and hydrophobic air bubbles generated during the agitation process. Consequently, higher concentrations of Tween 20 seem to play a crucial role in maintaining the structural integrity of the spike protein trimer, preventing undesired conformational changes induced by mechanical stress.

We explored the differences in spike conformation using HDX-MS. There were clear deuterium uptake differences upon agitation observed for formulation with 0.02% Tween 20, primarily affecting the S2 domain, with minor changes also detected in the S1 domain. Spike protein is known to undergo conformational changes where it adopts an open trimeric form when stored at cold temperatures (Costello et al.) [6]. The current HDX-MS data showed an increase in deuterium uptake upon agitation, mainly localized in the S2 stalk region, which could indicate further extension in the open trimeric form. This extended trimeric form was also consistent with the SEC-UPLC data, where an extended trimer was observed as an earlier eluting peak (Supplementary Figure S2). mAb 511 is a conformational epitope that binds to the S2 domain; its equivalent location in the D614 strain is in residues 969–977 [7]. However, there was minimal differential deuterium uptake observed in the S1 domain. This observation was supported by the ACE2 binding studies, shown in Figure 2, where there was no difference observed for the ACE2 binding pre- and post-agitation. Taken together, these results identify the S2 domain as the region that is impacted by agitation and that drives the change in conformation and extension of the trimer complex.

We do not suspect the spike protein is aggregating, as it has been demonstrated that the formation of higher-order aggregation is mainly driven by the S1 domain, specifically the C-terminal domain (CTD) of one spike protein interacting with the N-terminal domain (NTD) of another monomer [30,31]. In this study, the S1 domain was minimally affected compared to the S2 domain. Furthermore, aggregation would also be reflected in the SEC-UPLC data, where multiple higher Stokes radius species would be present, which was not observed in this study. We also believe that the open trimeric form adopted by the spike protein post-agitation may be of a different conformation compared to the ones published by Costello, S.M., et al. and Bruch, E.M., et al. [6,7]. The open trimeric form of the beta variant spike protein demonstrated by Eduardo et al. [7] resulted in the enhanced binding of anti-S2 (mAb 511) due to the exposure of the epitope and showed that the protein was able to reverse to its open trimer pre-fusion conformation. However, in this study, the D614 variant showed decreased binding to anti-S2 (mAb 511), and the conformational/structural change was not reversible. As such, we believe the spike protein may have adopted a misfolded extended trimeric form that was irreversible.

Predicting the shelf life and storage capability of a vaccine as early as possible is crucial to ensuring that the selected formulation can maintain its efficacy over time. Instead of waiting for extensive real-time stability studies, employing mathematical predictive modeling software allows for early comprehension and enhanced confidence in the chosen formulation. Additionally, kinetic predictive models can be useful for managing temperature excursions during cold chain breaks, thus further safeguarding the integrity of the vaccine. By utilizing advanced kinetic modeling techniques, our results have shown that spike protein formulated with 0.2% Tween remains stable for a minimum of two years under refrigerated conditions. This finding presents a significant advantage in terms of accessibility, cost-effectiveness, and logistics, especially when compared to mRNA vaccines, which typically require subzero storage to maintain stability [32]. Moreover, our results highlight an additional pivotal aspect: the formulation with 0.2% Tween demonstrates stability not only for the D614 variant but also for the B1351 variant of the spike protein. This finding indicates its potential to serve as a universal excipient for stabilizing spike protein, regardless of the variant.

Although Tween 20 effectively enhanced the colloidal stability of the spike protein, its effect on the protein’s thermal stability was negligible. Through the excipient screening using extrinsic fluorescence, we determined that sucrose and glycerol enhanced both the conformational and thermal stability of the spike protein. It is widely recognized that sugars and polyols play a pivotal role in improving stability by selectively excluding themselves from the protein’s surface, thereby promoting the hydration of the protein [33]. The formation of a more hydrated outer shell reinforces hydrophobic interactions and disfavors hydrophobic interactions between protein molecules through their surface hydrophobic patches, thereby preventing aggregation and augmenting the protein’s stability. The effects of sucrose on the thermal stability were confirmed using real-time stability studies, clearly indicating an increase in the stability of the spike protein at elevated temperatures. Even though 0.2% Tween 20 was sufficient to maintain the spike protein stability under refrigerated conditions and to improve robustness against mechanical stress, sucrose can provide the additional advantage of improving thermostability, which offers several benefits including expanded access to the vaccine, cost savings, and reduced waste.

In conclusion, our study not only provides valuable insights into the formulation of protein-based COVID-19 vaccines but also emphasizes the significance of surfactants and stabilizers in maintaining the structural and functional integrity of the spike protein, thereby contributing to the development of stable and effective vaccines for widespread use.

## Data Availability

All data that support this study are available from the corresponding author upon reasonable request.

## Supplementary Materials

The following supporting information can be downloaded at: https://www.mdpi.com/article/10.3390/vaccines12080830/s1, Figure S1: Effect of thermal stress at 37 °C ± 2 °C on spike protein (D614) at a concentration of 60 µg/mL containing 0.2% (*v*/*v*) Tween 20 in 10 mM PBS pH 7.0. Chromatographs are overlayed with T_0_ (purple), t5 day (brown) and t11 day (blue) thermal stress results. Spike protein (D614) was subjected to thermal stress in a 37 °C incubator for up to 11 days and tested for open trimer area, extended trimer area, and canonical trimer by SEC-UPLC; Figure S2: Effect of mechanical stress on spike protein (D614) at a concentration of 60 µg/mL containing 0.02% (*v*/*v*) Tween 20 in 10 mM PBS pH 7.0 (A), spike protein (D614) at a concentration of 60 µg/mL containing 0.2% (*v*/*v*) Tween 20 in 10 mM PBS pH 7.0 (B), and spike protein (B.1.351) at a concentration of 111 µg/mL containing 0.2% (*v*/*v*) Tween 20 in 10 mM PBS pH 7.0 (C). Chromatographs are overlayed with T_0_ (blue) and 24 h (red) mechanical stress results. Spike protein (D614 or B.1.351) was subjected to agitation stress in an orbital shaker at 300 rpm for 24 h and tested for open trimer area and extended trimer area by SEC-UPLC; Figure S3: The data for open trimer (Trimer 1) peak area (SEC-UPLC) and potency measured by ELISA trend together and correlated well for spike protein (D614) 60 µg/mL in 0.2% Tween 20 at 37 °C forced degradation temperature (*n* = 9, r2 = 0.978, *p* value < 0.0001); Figure S4: Effect of agitation stress (300 RMP for 24 h) on spike protein (D614) HOS in 10 mM PBS pH 7.0 formulation, containing 0.2% Tween 20 (panel A) or 0.02% Tween 20 (panel B), estimated by WSD of CD spectra (filled circles in panel C, left part) and intrinsic fluorescence spectra (filled circles in panel C, right part). The formulation containing Tween 20 at T_0_ was used as a reference for WSD calculation and the green bars show 1, 2, and 3 standard deviations (SD) for each sample; Figure S5: Long-term potency measured by ELISA of D614 variant at 60 µg/mL predicted by kinetic models (lines) at 5 °C (A), 25 °C (B), and 37 °C (C) for formulations containing 0.2% Tween 20 in PBS, with 30% sucrose (red) and without sucrose (blue). Data used for kinetic modeling are displayed as filled symbols. The ELISA predictions are shown with predictive band representing 95% PB (dashed lines). D—Time–temperature–transformation (TTT) diagrams from kinetic models of formulations with (red) and without (blue) 30% sucrose. Lines represent isoconversion from 1% to 90% for loss of antigenicity (ELISA). For both formulations, distinct degradation kinetics are identified by change in isoconversion slopes, around 37 °C; Table S1: Kinetic models.
