# Supplementary material for: Formulation Development of a COVID-19 Recombinant Spike Protein-Based Vaccine
Source: Vaccines (Basel). 2024 Jul 23;12(8):830. doi: 10.3390/vaccines12080830 (PMC11360652; doi:10.3390/vaccines12080830)
Supplement: Supplementary file 1 [file vaccines-12-00830-s001.zip › vaccines-3081517-supplementary.pdf]

Supplementary Materials

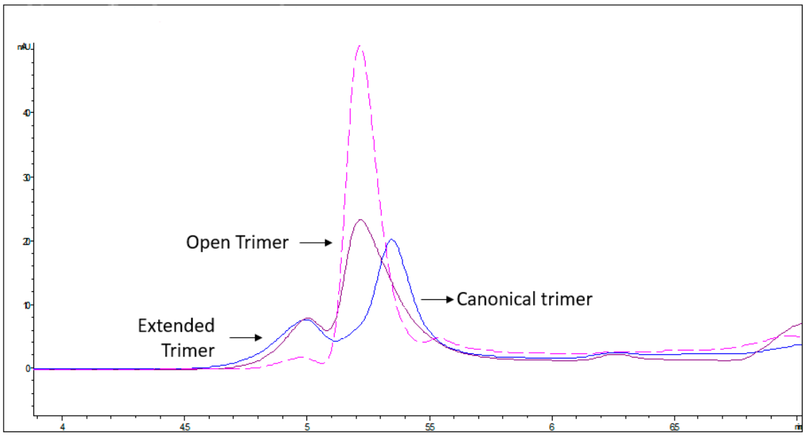

Supplementary Figure S1: Effect of thermal stress at 37°C ± 2°C on spike protein (D614) at a concentration of 60 µg/mL containing 0.2% (v/v) Tween 20 in 10 mM PBS pH 7.0. Chromatographs are overlayed with T_0 (Purple), t5 day (Brown) and t11 day (Blue) thermal stress results. Spike protein (D614) was subjected to thermal stress in a 37°C incubator for up to 11 days and tested for open trimer area, extended trimer area and canonical trimer by SEC-UPLC.

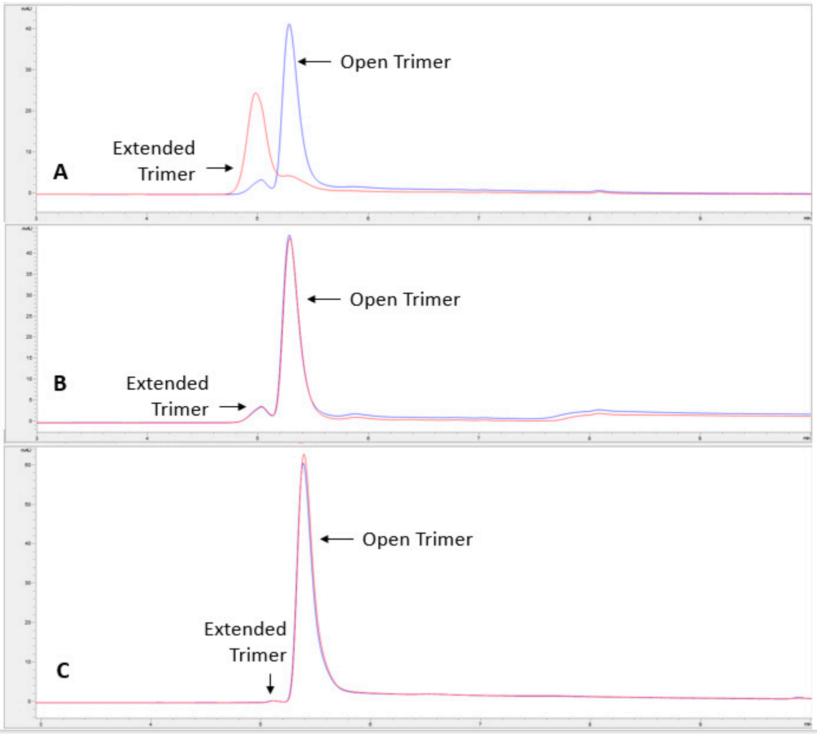

Supplementary Figure S2: Effect of mechanical stress on spike protein (D614) at a concentration of 60 µg/mL containing 0.02% (v/v) Tween 20 in 10 mM PBS pH 7.0 (A), spike protein (D614) at a concentration of 60 µg/mL containing 0.2% (v/v) Tween 20 in 10 mM PBS pH 7.0 (B) and spike protein (B.1.351) at a concentration of 111 µg/mL containing 0.2% (v/v) Tween 20 in 10 mM PBS pH 7.0 (C). Chromatographs are overlayed with T_0 (Blue) and 24 hr (Red) mechanical stress results. Spike protein (D614 or B.1.351) was subjected to agitation stress in an orbital shaker at 300 rpm for 24 h and tested for open trimer area and extended trimer area by SEC-UPLC.

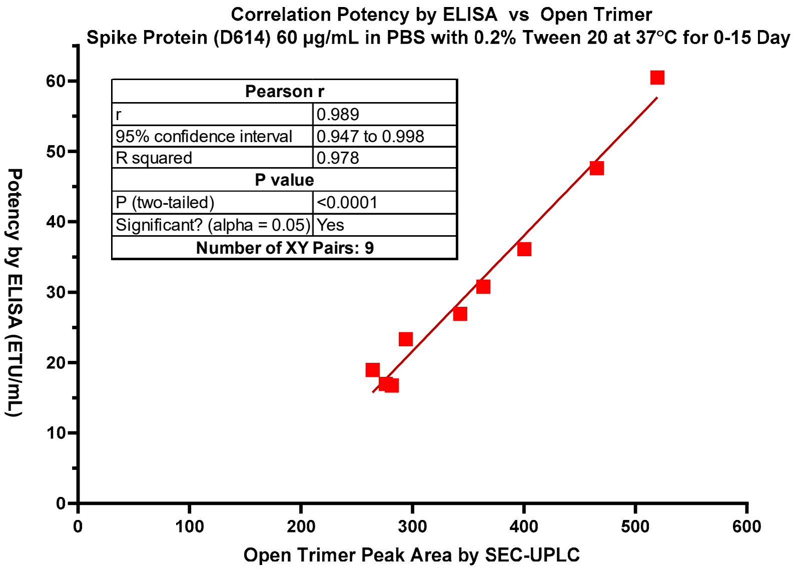

Supplementary Figure S3: The data for Open Trimer (Trimer 1) Peak Area (SEC-UPLC) and Potency by ELISA trend together and correlated well for spike protein (D614) 60 µg/mL in 0.2% Tween 20 at 37°C forced degradation temperature (n=9, r2=0.978, p value <0.0001).

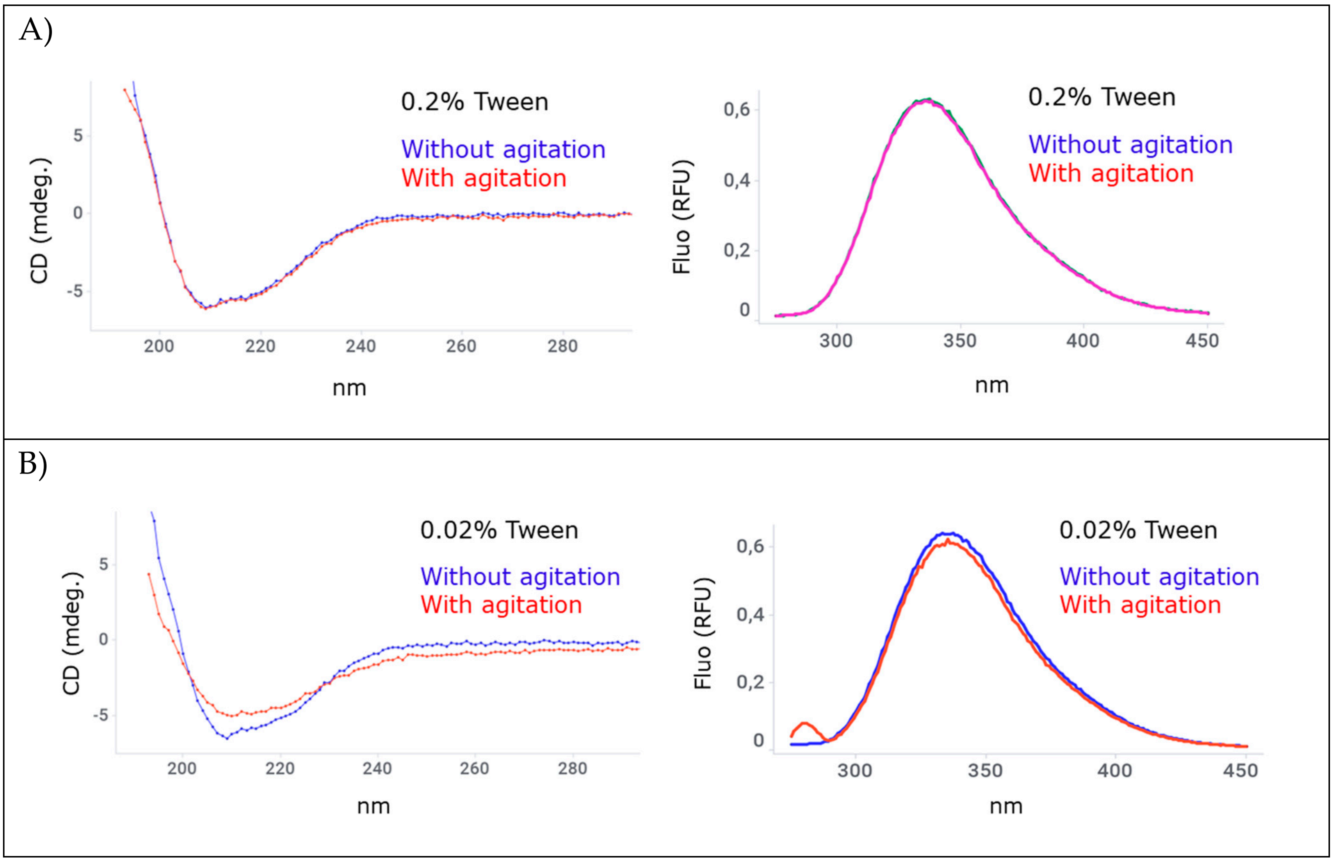

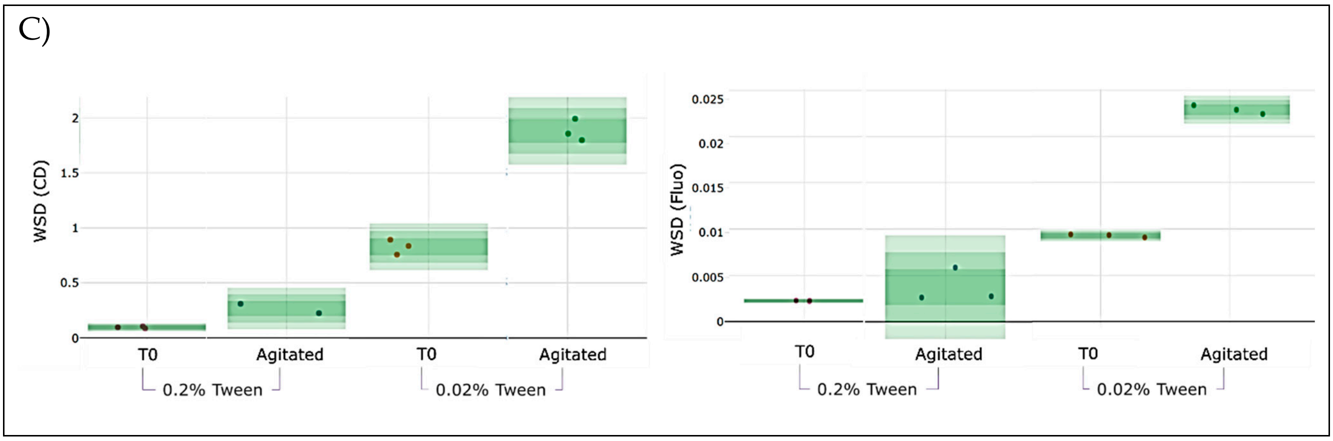

Supplementary Figure S4: Effect of agitation stress (300 RMP for 24 h) on spike protein (D614) HOS in 10 mM PBS pH 7.0 formulation, containing 0.2% Tween 20 (panel A) or 0.02% Tween 20 (panel B), estimated by WSD of CD spectra (filled circles in panel C, left part) and intrinsic fluorescence spectra (filled circles in panel C, right part). The formulation containing Tween 20 at T_0 was used as a reference for WSD calculation and the green bars show 1, 2 and 3 standard deviations (SD) for each sample.

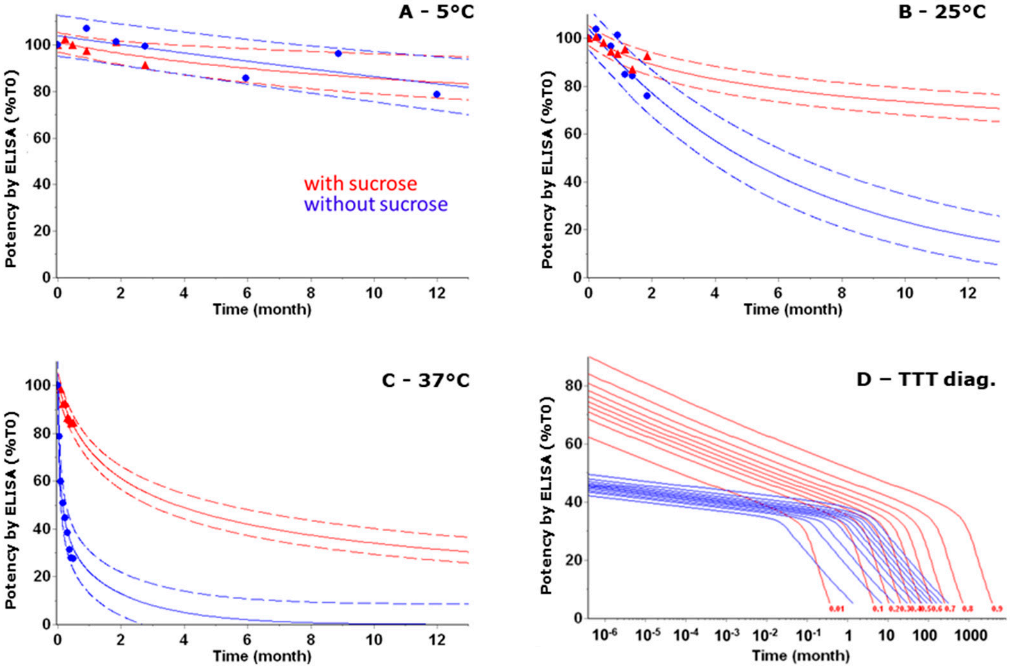

Supplementary Figure S5: Long-term Potency by ELISA of D614 variant at 60 µg/mL predicted by kinetic models (lines) at 5°C (A), 25°C (B) and 37°C (C) for formulations containing 0.2 % Tween 20 in PBS, with 30% sucrose (red) and without sucrose (blue). Data used for kinetic modeling are displayed as filled symbols. The ELISA predictions are shown with predictive band representing 95% PB (dashed lines). D - Time-temperature-transformation (TTT) diagrams from kinetic models of formulations with (red) and without (blue) 30% sucrose. Lines represent isoconversion from 1% to 90% for loss of antigenicity (ELISA). For both formulations, distinct degradation kinetics are identified by change of isoconversion slopes, around 37°C.

Supplementary Table S1: Kinetic models

		Equation
A	**Kinetic models, leading for D614 variants to a two-step model (Applied in** Error! Reference source not found.**A)**	$\frac{d\alpha}{dt} = 0.89 \times \exp(63.9) \times \exp\left(-\frac{200.8E3}{RT}\right).(1-\alpha)^1 + 0.11 \times \exp(5.8) \times \exp\left(-\frac{49.1E3}{RT}\right).(1-\alpha)^3$
B	**Kinetic models, leading for B.1351 variants to a mixture of a one-step and a two-step model (Applied in** Error! Reference source not found.**B)**	**One-step model (wAIC / wBIC = 89.3% / 75.0%):** $\frac{d\alpha}{dt} = \exp(139.2) \times \exp\left(-\frac{379.8E3}{RT}\right).(1-\alpha)^8$ **Two-step model (wAIC / wBIC = 10.7% / 25.0%):** $\frac{d\alpha}{dt} = 0.44 \times \exp(62.6) \times \exp\left(-\frac{190.5E3}{RT}\right) \times (1-\alpha_1)^1 + 0.56 \times \exp(371.9) \times \exp\left(-\frac{100.0E3}{RT}\right)$
C	**Kinetic model for D614 variant formulation containing 0.2% (v/v) Tween 20 with sucrose (Applied in** Supplementary **Figure S4)**	$\frac{d\alpha}{dt} = \exp(-2.6) \times \exp\left(-\frac{363.6E3}{RT}\right).(1-\alpha)^{3.4} + \exp(131.2) \times \exp\left(-\frac{378.9E3}{RT}\right).(1-\alpha)^4$
D	**Kinetic model for D614 variant formulation containing 0.2% (v/v) Tween 20 without sucrose (Applied in** Supplementary **Figure S4)**	$\frac{d\alpha}{dt} = \exp(24.4) \times \exp\left(-\frac{102.0E3}{RT}\right).(1-\alpha)^1 + \exp(444.3) \times \exp\left(-\frac{117.7E3}{RT}\right).(1-\alpha)^4$
